# On modified Dunkl generalization of Szász operators via *q*-calculus

**DOI:** 10.1186/s13660-017-1311-5

**Published:** 2017-02-07

**Authors:** M Mursaleen, Md Nasiruzzaman, Abdullah Alotaibi

**Affiliations:** 1 Department of Mathematics, Aligarh Muslim University, Aligarh, 202002 India; 2 Operator Theory and Applications Research Group, Department of Mathematics, Faculty of Science, King Abdulaziz University, P.O. Box 80203, Jeddah, 21589 Saudi Arabia

**Keywords:** 41A25, 41A36, 33C45, *q*-integers, Dunkl analogue, Szász operator, *q*-Szász-Mirakjan-Kantorovich, modulus of continuity, Peetre’s *K*-functional

## Abstract

The purpose of this paper is to introduce a modification of *q*-Dunkl generalization of exponential functions. These types of operators enable better error estimation on the interval $[\frac{1}{2},\infty)$ than the classical ones. We obtain some approximation results via a well-known Korovkin-type theorem and a weighted Korovkin-type theorem. Further, we obtain the rate of convergence of the operators for functions belonging to the Lipschitz class.

## Introduction and preliminaries

In 1912, Bernstein [1] introduced the following sequence of operators $B_{n}:C[0,1]\rightarrow C[0,1]$ defined by

1.1 $$ B_{n}(f;x)=\sum_{k=0}^{n} \binom{n}{k}x^{k}(1-x)^{n-k}f \biggl( \frac{k}{n} \biggr) , \quad x\in{}[0,1] $$

for $n\in\mathbb{N}$ and $f\in C[0,1]$.

In 1950, for $x \geq0$, Szász [2] introduced the operators

1.2 $$ S_{n}(f;x)=e^{-nx}\sum _{k=0}^{\infty}\frac{(nx)^{k}}{k!} f \biggl( \frac{k}{n} \biggr),\quad f \in C[0,\infty). $$

In the field of approximation theory, the application of *q*-calculus emerged as a new area. The first *q*-analogue of well-known Bernstein polynomials was introduced by Lupaş by applying the idea of *q*-integers [3]. In 1997, Phillips [4] considered another *q*-analogue of the classical Bernstein polynomials. Later on, many authors introduced *q*-generalizations of various operators and investigated several approximation properties [5–14].

We now present some basic definitions and notations of the *q*-calculus which are used in this paper [15].

### Definition 1.1

For $|q|<1$, the *q*-number $[ \lambda ] _{q} $ is defined by

1.3 $$ [\lambda ]_{q}=\left \{ \textstyle\begin{array}{l@{\quad}l} \frac{1-q^{\lambda}}{1-q} & (\lambda\in\mathbb{C}), \\ \sum_{k=0}^{n-1}q^{k}=1+q+q^{2}+\cdots+q^{n-1} & (\lambda=n\in \mathbb{N}).\end{array}\displaystyle \right . $$

### Definition 1.2

For $|q|<1$, the *q*-factorial $[ n ] _{q}!$ is defined by

1.4 $$ [ n ] _{q}!=\left \{ \textstyle\begin{array}{l@{\quad}l} 1 & (n=0), \\ \prod_{k=1}^{n} [ k ] _{q} & (n\in\mathbb{N}).\end{array}\displaystyle \right . $$

Our investigation is to construct a linear positive operator generated by a generalization of the exponential function defined by (see [16])

$$ e_{\mu}(x)=\sum_{n=0}^{\infty} \frac{x^{n}}{\gamma_{\mu}(n)}, $$

where

$$ \gamma_{\mu}(2k)=\frac{2^{2k}k!\Gamma ( k+\mu+\frac{1}{2} ) }{\Gamma ( \mu+\frac{1}{2} ) }, $$

and

$$ \gamma_{\mu}(2k+1)=\frac{2^{2k+1}k!\Gamma ( k+\mu+\frac {3}{2} ) }{\Gamma ( \mu+\frac{1}{2} ) }. $$

The recursion formula for $\gamma_{\mu}$ is given by

$$ \gamma_{\mu}(k+1)=(k+1+2\mu\theta_{k+1})\gamma_{\mu }(k), \quad k=0,1,2,\ldots, $$

where $\mu>-\frac{1}{2}$ and

$$ \theta_{k}=\left \{ \textstyle\begin{array}{l@{\quad}l} 0 & \text{if }k\in2\mathbb{N}, \\ 1 & \text{if }k\in2\mathbb{N}+1.\end{array}\displaystyle \right . $$

Sucu [17] defined a Dunkl analogue of Szász operators via a generalization of the exponential function [16] as follows:

1.5 $$ S_{n}^{\ast}(f;x):=\frac{1}{e_{\mu}(nx)}\sum _{k=0}^{\infty}\frac {(nx)^{k}}{\gamma_{\mu}(k)}f \biggl( \frac{k+2\mu\theta_{k}}{n} \biggr) , $$

where $x\geq0$, $f\in C[0,\infty)$, $\mu\geq0$, $n\in\mathbb{N}$.

Cheikh *et al.* [18] stated the *q*-Dunkl classical *q*-Hermite-type polynomials and gave definitions of *q*-Dunkl analogues of exponential functions and recursion relations for $\mu>-\frac{1}{2}$ and $0< q<1$,

1.6 $$\begin{aligned}& e_{\mu,q}(x)=\sum_{n=0}^{\infty} \frac{x^{n}}{\gamma_{\mu,q}(n)}, \quad x\in {}[0,\infty), \end{aligned}$$

1.7 $$\begin{aligned}& E_{\mu,q}(x)=\sum_{n=0}^{\infty} \frac{q^{\frac {n(n-1)}{2}}x^{n}}{\gamma _{\mu,q}(n)}, \quad x\in{}[0,\infty), \end{aligned}$$

where

1.8 $$ \gamma_{\mu,q}(n)=\frac{(q^{2\mu+1},q^{2})_{[\frac{n+1}{2}]}(q^{2},q^{2})_{[\frac{n}{2}]}}{(1-q)^{n}},\quad n\in\mathbb{N}. $$

Some of the special cases of $\gamma_{\mu,q}(n)$ are defined as follows:

$$\begin{aligned}& \gamma_{\mu,q}(0)=1,\qquad \gamma_{\mu,q}(1)=\frac{1-q^{2\mu+1}}{1-q}, \\& \gamma_{\mu,q}(2)= \biggl(\frac{1-q^{2\mu+1}}{1-q} \biggr) \biggl( \frac {1-q^{2}}{1-q} \biggr), \\& \gamma_{\mu,q}(3)= \biggl(\frac{1-q^{2\mu+1}}{1-q} \biggr) \biggl( \frac {1-q^{2}}{1-q} \biggr) \biggl(\frac{1-q^{2\mu+3}}{1-q} \biggr), \\& \gamma_{\mu,q}(4)= \biggl(\frac{1-q^{2\mu+1}}{1-q} \biggr) \biggl( \frac {1-q^{2}}{1-q} \biggr) \biggl(\frac{1-q^{2\mu+3}}{1-q} \biggr) \biggl( \frac {1-q^{4}}{1-q} \biggr). \end{aligned}$$

In [19], Içöz and Çekim gave the Dunkl generalization of Szász operators via *q*-calculus as follows:

1.9 $$ D_{n,q}(f;x)=\frac{1}{e_{\mu,q}([n]_{q}x)}\sum_{k=0}^{\infty} \frac{([n]_{q}x)^{k}}{\gamma_{\mu,q}(k)}f \biggl( \frac{1-q^{2\mu\theta _{k}+k}}{1-q^{n}} \biggr) $$

for $\mu>\frac{1}{2}$, $x\geq0$, $0< q<1$ and $f\in C[0,\infty)$.

Previous studies demonstrate that providing a better error estimation for positive linear operators plays an important role in approximation theory, which allows us to approximate much faster to the function being approximated.

Motivated essentially by Içöz and Çekim’s [19] recent investigation of Dunkl generalization of Szász-Mirakjan operators via *q*-calculus, we show that our modified operators have better error estimation than those in [19]. We also prove several approximation results and successfully extend the results of [19]. Several other related results are also discussed.

## Construction of operators and moments estimation

Let $\{r_{[n]_{q}}\}$ be a sequence of real-valued continuous functions defined on $[0,\infty)$ with $0\leq r_{[n]_{q}}(x)<\infty$ such that

2.1 $$ r_{[n]_{q}}(x)=x-\frac{1}{2[n]_{q}}, \quad \mbox{where } \frac{1}{2n} \leq x< \frac{1}{1-q^{n}} \mbox{ and } n\in\mathbb{N}. $$

Then, for any $\frac{1}{2n} \leq x < \frac{1}{1-q^{n}}$, $0< q<1$, $\mu>\frac{ 1}{2n}$ and $n \in\mathbb{N}$, we define

2.2 $$ D_{n,q}^{\ast}(f;x)=\frac{1}{e_{\mu,q}([n]_{q}r_{[n]_{q}}(x))}\sum _{k=0}^{\infty}\frac{([n]_{q}r_{[n]_{q}}(x))^{k}}{\gamma_{\mu ,q}(k)}f \biggl( \frac{1-q^{2\mu\theta_{k}+k}}{1-q^{n}} \biggr) , $$

where $e_{\mu,q}(x)$, $\gamma_{\mu,q}$ are defined in (1.6), (1.8) by [17] and $f\in C_{\zeta}[0,\infty)$ with $\zeta \geq0$ and

2.3 $$ C_{\zeta}[0,\infty)=\bigl\{ f\in C[0,\infty):\bigl\vert f(t)\bigr\vert \leq M(1+t)^{\zeta } \mbox{ for some } M>0, \zeta>0\bigr\} . $$

### Lemma 2.1

*Let* $D_{n,q}^{\ast}(\cdot ; \cdot)$ *be the operators given by* (2.2). *Then*, *for each* $\frac{1}{2n}\leq x<\frac{1}{1-q^{n}}$, $n\in \mathbb{N}$, *we have the following identities*/*estimates*:

1. $D_{n,q}^{\ast}(1;x)=1$,
2. $D_{n,q}^{\ast}(t;x)=x-\frac{1}{2[n]_{q}}$,
3. $x^{2}+ ( q^{2 \mu}[1-2 \mu]_{q} \frac{e_{\mu,q}(q [n]_{q} r_{[n]_{q}}(x))}{e_{\mu,q}([n]_{q} r_{[n]_{q}} (x))}-1 )\frac {x}{[n]_{q}}-\frac{1}{4[n]_{q}^{2}} (2q^{2 \mu}[1-2 \mu]_{q} \frac{e_{\mu,q}(q [n]_{q} r_{[n]_{q}}(x))}{e_{\mu,q}([n]_{q} r_{[n]_{q}} (x))}-1 )\leq D_{n,q}^{\ast}(t^{2};x) \leq x^{2}+ ( [1+2 \mu]_{q} -1 )\frac {x}{[n]_{q}}-\frac{1}{4[n]_{q}^{2}} (2[1+2 \mu]_{q} -1 ) $.

### Proof

As $D_{n,q}^{\ast}(1;x)=\frac{1}{e_{\mu,q}([n]_{q}r_{[n]_{q}}(x))}\sum_{k=0}^{\infty}\frac{([n]_{q}r_{[n]_{q}}(x))^{k}}{\gamma_{\mu }(k)}=1$, and

$$\begin{aligned} D_{n,q}^{\ast}(t;x) =&\frac{1}{e_{\mu,q}([n]_{q}r_{[n]_{q}}(x))}\sum _{k=0}^{\infty}\frac{([n]_{q}r_{[n]_{q}}(x))^{k}}{\gamma_{\mu }(k)} \biggl( \frac{1-q^{2\mu\theta_{k}+k}}{1-q^{n}} \biggr) \\ =&\frac{1}{[n]_{q}e_{\mu,q}([n]_{q}r_{[n]_{q}}(x))}\sum_{k=1}^{\infty }\frac{([n]_{q}r_{[n]_{q}}(x))^{k}}{\gamma_{\mu}(k-1)} \\ =&x-\frac{1}{2[n]_{q}}, \end{aligned}$$

then (1) and (2) hold. Similarly,

$$\begin{aligned} D_{n,q}^{\ast}\bigl(t^{2};x\bigr) =& \frac{1}{e_{\mu,q}([n]_{q}r_{[n]_{q}}(x))}\sum_{k=0}^{\infty} \frac{([n]_{q}r_{[n]_{q}}(x))^{k}}{\gamma_{\mu }(k)} \biggl( \frac{1-q^{2\mu\theta_{k}+k}}{1-q^{n}} \biggr) ^{2} \\ =&\frac{1}{[n]_{q}^{2}e_{\mu,q}([n]_{q}r_{[n]_{q}}(x))}\sum_{k=0}^{\infty}\frac{([n]_{q}r_{[n]_{q}}(x))^{k}}{\gamma_{\mu}(k-1)} \biggl( \frac{1-q^{2\mu\theta_{k}+k}}{1-q} \biggr) \\ =&\frac{1}{[n]_{q}^{2}e_{\mu,q}([n]_{q}r_{[n]_{q}}(x))}\sum_{k=0}^{\infty}\frac{([n]_{q}r_{[n]_{q}}(x))^{k+1}}{\gamma_{\mu}(k)} \biggl( \frac{1-q^{2\mu\theta_{k+1}+k+1}}{1-q} \biggr) . \end{aligned}$$

From [19] we know that

2.4 $$ [2\mu\theta_{k+1}+k+1]_{q}=[2\mu\theta_{k}+k]_{q}+q^{2\mu \theta _{k}+k} \bigl[2\mu(-1)^{k}+1\bigr]_{q}. $$

Now, by separating to the even and odd terms and using (2.4), we get

$$\begin{aligned} D_{n,q}^{\ast}\bigl(t^{2};x\bigr) =& \frac{1}{[n]_{q}^{2}e_{\mu ,q}([n]_{q}r_{[n]_{q}}(x))}\sum_{k=0}^{\infty} \frac{([n]_{q}r_{[n]_{q}}(x))^{k+1}}{\gamma_{\mu}(k)} \biggl( \frac{1-q^{2\mu \theta_{k+1}+k+1}}{1-q} \biggr) \\ &{}+\frac{[1+2\mu]_{q}}{[n]_{q}^{2}e_{\mu,q}([n]_{q}r_{[n]_{q}}(x))}\sum_{k=0}^{\infty} \frac{([n]_{q}r_{[n]_{q}}(x))^{2k+1}}{\gamma_{\mu }(2k)}q^{2\mu\theta_{2k}+2k} \\ &{}+\frac{[1-2\mu]_{q}}{[n]_{q}^{2}e_{\mu,q}([n]_{q}r_{[n]_{q}}(x))}\sum_{k=0}^{\infty} \frac{([n]_{q}r_{[n]_{q}}(x))^{2k+2}}{\gamma_{\mu }(2k)}q^{2\mu\theta_{2k+1}+2k+1}. \end{aligned}$$

Since

2.5 $$ {}[1-2\mu]_{q}\leq{}[1+2\mu]_{q}, $$

we have

$$\begin{aligned} D_{n,q}^{\ast}\bigl(t^{2};x\bigr) \geq& \bigl(r_{[n]_{q}}(x)\bigr)^{2}+\frac{r_{[n]_{q}}(x)[1-2\mu]_{q}}{[n]_{q}e_{\mu,q}([n]_{q}r_{[n]_{q}}(x))}\sum _{k=0}^{\infty}\frac{(q[n]_{q}r_{[n]_{q}}(x))^{2k}}{\gamma_{\mu}(2k)} \\ &{}+\frac{q^{2\mu}r_{[n]_{q}}(x)[1-2\mu]_{q}}{[n]_{q}e_{\mu ,q}([n]_{q}r_{[n]_{q}}(x))}\sum_{k=0}^{\infty} \frac{(q[n]_{q}r_{[n]_{q}}(x))^{2k+1}}{\gamma_{\mu}(2k+1)} \\ \geq&\bigl(r_{[n]_{q}}(x)\bigr)^{2}+q^{2\mu}[1-2 \mu]_{q}\frac{e_{\mu ,q}(q[n]_{q}r_{[n]_{q}}(x))}{e_{\mu,q}([n]_{q}r_{[n]_{q}}(x))}\frac{r_{[n]_{q}}(x)}{[n]_{q}}. \end{aligned}$$

On the other hand, we have

$$ D_{n,q}^{\ast}\bigl(t^{2};x\bigr)\leq \bigl(r_{[n]_{q}}(x)\bigr)^{2}+[1+2\mu]_{q} \frac{r_{[n]_{q}}(x)}{[n]_{q}}. $$

This completes the proof. □

### Lemma 2.2

*Let the operators* $D_{n,q}^{\ast}(\cdot ; \cdot)$ *be given by* (2.2). *Then*, *for each* $x\geq\frac{1}{2n}$, $n \in\mathbb{N} $, *we have*

1. $D_{n,q}^{\ast}(t-x;x)=-\frac{1}{2 [n]_{q}}$,
2. $D_{n,q}^{\ast}((t-x)^{2};x)\leq[1+2\mu ]_{q}\frac{x}{[n]_{q}}-\frac{1}{4[n]_{q}^{2}} ( 2[1+2\mu]_{q}-1 ) $.

### Proof

For the proof of this lemma, we use Lemma 2.1. In view of

$$ D_{n}^{\ast}(t-x;x)=D_{n}^{\ast}(t;x)-D_{n}^{\ast}(1;x), $$

(1) follows immediately.

Also

$$\begin{aligned} D_{n}^{\ast}\bigl((t-x)^{2};x\bigr) =&D_{n}^{\ast}\bigl(t^{2};x\bigr)-2xD_{n}^{\ast }(t;x)+x^{2}D_{n}^{\ast}(1;x) \\ \leq&x^{2}+ \bigl( [1+2\mu]_{q}-1 \bigr) \frac{x}{[n]_{q}}-\frac{1}{4[n]_{q}^{2}} \bigl( 2[1+2\mu]_{q}-1 \bigr) \\ &{}-2x \biggl( x-\frac {1}{2[n]_{q}} \biggr) +x^{2} \\ \leq&[1+2\mu]_{q}\frac{x}{[n]_{q}}-\frac{1}{4[n]_{q}^{2}} \bigl( 2[1+2 \mu ]_{q}-1 \bigr) . \end{aligned}$$

This proves (2). □

## Main results

We obtain the Korovkin-type approximation properties for our operators (see [20–22]).

Let $C_{B}(\mathbb{R}^{+})$ be the set of all bounded and continuous functions on $\mathbb{R}^{+}=[0,\infty)$, which is a linear normed space with

$$ \| f\|_{C_{B}}=\sup_{x\geq0}\bigl\vert f(x)\bigr\vert . $$

Let

$$ H:=\biggl\{ f:x\in{}[0,\infty),\frac{f(x)}{1+x^{2}} \mbox{ is convergent as } x \rightarrow\infty\biggr\} . $$

### Theorem 3.1

*Let* $D_{n,q}^{\ast}(\cdot ; \cdot)$ *be the operators defined by* (2.2). *Then*, *for any function* $f\in C_{\zeta}[0,\infty)\cap H$, $\zeta\geq2$,

$$ \lim_{n\rightarrow\infty}D_{n,q}^{\ast}(f;x)=f(x) $$

*is uniform on each compact subset of* $[0,\infty)$, *where* $x \in[ \frac{1}{2},b)$, $b>\frac{1}{2}$.

### Proof

The proof is based on Lemma 2.1 and the well-known Korovkin theorem regarding the convergence of a sequence of linear positive operators, so it is enough to prove the conditions

$$ \lim_{n\rightarrow\infty}D_{n,q}^{\ast}\bigl(t^{j};x \bigr)=x^{j},\quad j=0,1,2\ (\mbox{as } n\rightarrow\infty) $$

uniformly on $[0,1]$.

Clearly, $\frac{1}{[n]_{q}}\rightarrow0$ ($n\rightarrow\infty$) we have

$$ \lim_{n\rightarrow\infty}D_{n,q}^{\ast}(t;x)=x, \qquad \lim _{n\rightarrow \infty}D_{n,q}^{\ast}\bigl(t^{2};x \bigr)=x^{2}. $$

This completes the proof. □

We recall the weighted spaces of the functions on $\mathbb{R}^{+}$, which are defined as follows:

$$\begin{aligned}& P_{\rho}\bigl(\mathbb{R}^{+}\bigr) = \bigl\{ f:\bigl\vert f(x)\bigr\vert \leq M_{f}\rho (x) \bigr\} , \\& Q_{\rho}\bigl(\mathbb{R}^{+}\bigr) = \bigl\{ f:f\in P_{\rho}\bigl(\mathbb {R}^{+}\bigr)\cap C[0,\infty) \bigr\} , \\& Q_{\rho}^{k}\bigl(\mathbb{R}^{+}\bigr) = \biggl\{ f:f\in Q_{\rho}\bigl(\mathbb {R}^{+}\bigr) \mbox{ and } \lim_{x\rightarrow\infty}\frac{f(x)}{\rho(x)}=k\ (k \mbox{ is a constant}) \biggr\} , \end{aligned}$$

where $\rho(x)=1+x^{2}$ is a weight function and $M_{f}$ is a constant depending only on *f*. Note that $Q_{\rho}(\mathbb{R}^{+})$ is a normed space with the norm $\| f\|_{\rho}=\sup_{x\geq0}\frac {|f(x)|}{\rho(x)}$.

### Lemma 3.2

[23]

*The linear positive operators* $L_{n}$, $n\geq1$ *act from* $Q_{\rho}(\mathbb{R}^{+})\to P_{\rho}(\mathbb{R}^{+}) $ *if and only if*

$$ \bigl\Vert L_{n}(\varphi;x) \bigr\Vert \leq K\varphi(x), $$

*where* $\varphi(x)=1+x^{2}$, $x \in\mathbb{R}^{+}$ *and* *K* *is a positive constant*.

### Theorem 3.3

[23]

*Let* $\{L_{n}\}_{n \geq1}$ *be a sequence of positive linear operators acting from* $Q_{\rho}(\mathbb{R}^{+})\to P_{\rho}(\mathbb{R}^{+}) $ *and satisfying the condition*

$$ \lim_{n\to\infty} \bigl\Vert L_{n}\bigl( \rho^{\tau}\bigr)-\rho^{\tau}\bigr\Vert _{\varphi}=0, \quad \tau=0,1,2. $$

*Then*, *for any function* $f\in Q_{\rho}^{k}(\mathbb{R}^{+})$, *we have*

$$ \lim_{n\to\infty} \bigl\Vert L_{n}(f;x)-f \bigr\Vert _{\varphi}=0. $$

### Theorem 3.4

*Let* $D_{n,q}^{\ast}(\cdot ; \cdot)$ *be the operators defined by* (2.2). *Then*, *for each function* $f \in Q^{k}_{\rho}(\mathbb{R}^{+})$, *we have*

$$ \lim_{n\to\infty} \bigl\Vert D_{n,q}^{\ast}(f;x)-f \bigr\Vert _{\rho}=0. $$

### Proof

From Lemma 2.1 and Theorem 3.3, for $\tau=0$, the first condition is fulfilled. Therefore,

$$ \lim_{n\rightarrow\infty}\bigl\Vert D_{n,q}^{\ast}(1;x)-1 \bigr\Vert _{\rho }=0. $$

Similarly, from Lemma 2.1 and Theorem 3.3, for $\tau =1,2$ we have that

$$\begin{aligned} \sup_{x\in{}[0,\infty)}\frac{| D_{n,q}^{\ast}(t;x)-x| }{1+x^{2}} \leq&\frac{1}{2[n]_{q}}\sup _{x\in{}[0,\infty)}\frac {1}{1+x^{2}} \\ =&\frac{1}{2[n]_{q}}, \end{aligned}$$

which implies that

$$\begin{aligned}& \lim_{n\rightarrow\infty}\bigl\Vert D_{n,q}^{\ast}(t;x)-x \bigr\Vert _{\rho }=0, \\& \sup_{x\in{}[0,\infty)}\frac{| D_{n,q}^{\ast }(t^{2};x)-x^{2}| }{1+x^{2}} \leq\frac{|{}[1+2\mu]_{q}-1|}{[n]_{q}}\sup _{x\in {}[0,\infty)}\frac{x}{1+x^{2}} \\& \hphantom{\sup_{x\in{}[0,\infty)}\frac{| D_{n,q}^{\ast }(t^{2};x)-x^{2}| }{1+x^{2}} \leq{}}{}+\frac{1}{4[n]_{q}^{2}}\bigl\vert [1+2\mu]_{q}-1\bigr\vert \sup_{x\in {}[ 0,\infty)}\frac{1}{1+x^{2}}. \end{aligned}$$

Hence

$$ \lim_{n\rightarrow\infty}\bigl\Vert D_{n,q}^{\ast } \bigl(t^{2};x\bigr)-x^{2}\bigr\Vert _{\rho}=0. $$

This completes the proof. □

## Rate of convergence

Let $f\in C_{B}[0,\infty]$, the space of all bounded and continuous functions on $[0,\infty)$ and $x\geq\frac{1}{2n}$, $n\in\mathbb{N}$. Then, for $\delta>0$, the modulus of continuity of *f* denoted by $\omega (f,\delta)$ gives the maximum oscillation of *f* in any interval of length not exceeding $\delta>0$, and it is given by

4.1 $$ \omega(f,\delta)=\sup_{| t-x|\leq\delta}\bigl\vert f(t)-f(x)\bigr\vert , \quad t\in{}[0,\infty). $$

It is known that $\lim_{\delta\rightarrow0+}\omega(f,\delta)=0$ for $f\in C_{B}[0,\infty)$, and for any $\delta>0$ we have

4.2 $$ \bigl\vert f(t)-f(x)\bigr\vert \leq \biggl( \frac{| t-x|}{\delta}+1 \biggr) \omega (f,\delta). $$

Now we calculate the rate of convergence of operators (2.2) by means of modulus of continuity and Lipschitz-type maximal functions.

### Theorem 4.1

*Let* $D_{n,q}^{\ast}(\cdot ; \cdot)$ *be the operators defined by* (2.2). *Then*, *for* $f\in C_{B}[0,\infty)$, $x\geq\frac{1}{2n}$ *and* $n\in\mathbb{N}$, *we have*

$$ \bigl\vert D_{n,q}^{\ast}(f;x)-f(x)\bigr\vert \leq2\omega ( f;\delta _{n,x} ) , $$

*where*

4.3 $$ \delta_{n,x}=\sqrt{[1+2\mu]_{q}\frac{x}{[n]_{q}}- \frac {1}{4[n]_{q}^{2}} \bigl( 2[1+2\mu]_{q}-1 \bigr) }. $$

### Proof

We prove it by using (4.1), (4.2) and the Cauchy-Schwarz inequality. We can easily get

$$ \bigl\vert D_{n,q}^{\ast}(f;x)-f(x)\bigr\vert \leq \biggl\{ 1+\frac{1}{\delta} \bigl( D_{n,q}^{\ast}(t-x)^{2};x \bigr) ^{\frac{1}{2}} \biggr\} \omega (f;\delta) $$

if we choose $\delta=\delta_{n,x}$, and by applying the result (2) of Lemma 2.2, we get the result. □

### Remark 4.2

For the operators $D_{n,q}(\cdot ; \cdot)$ defined by (1.9) we may write that, for every $f\in C_{B}[0,\infty)$, $x\geq0$ and $n \in\mathbb{N}$,

4.4 $$ \bigl\vert D_{n,q}(f;x)-f(x)\bigr\vert \leq2\omega (f;\lambda_{n,x} ), $$

where by [19] we have

4.5 $$ \lambda_{n,x}=\sqrt{D_{n,q} \bigl((t-x)^{2};x\bigr)}\leq\sqrt{[1+2\mu]_{q} \frac{x}{[n]_{q}}}. $$

Now we claim that the error estimation in Theorem 4.1 is better than that of (4.4) provided $f\in C_{B}[0,\infty)$ and $x\geq\frac {1}{2n}$, $n\in\mathbb{N}$. Indeed, for $x\geq\frac{1}{2n}$, $\mu\geq\frac {1}{2n}$ and $n\in\mathbb{N}$, it is guaranteed that

4.6 $$\begin{aligned}& D_{n,q}^{\ast}\bigl((t-x)^{2};x\bigr)\leq D_{n,q}\bigl((t-x)^{2};x\bigr), \end{aligned}$$

4.7 $$\begin{aligned}& {}[1+2\mu]_{q}\frac{x}{[n]_{q}}-\frac{1}{4[n]_{q}^{2}} \bigl( 2[1+2\mu ]_{q}-1 \bigr) \leq{}[1+2\mu]_{q}\frac{x}{[n]_{q}}, \end{aligned}$$

which implies that

4.8 $$ \sqrt{[1+2\mu]_{q}\frac{x}{[n]_{q}}-\frac{1}{4[n]_{q}^{2}} \bigl( 2[1+2\mu]_{q}-1 \bigr) }\leq\sqrt{[1+2\mu]_{q} \frac{x}{[n]_{q}}}. $$

Now we give the rate of convergence of the operators ${D}_{n,q}^{\ast }(f;x) $ defined in (2.2) in terms of the elements of the usual Lipschitz class $\operatorname{Lip}_{M}(\nu)$.

Let $f\in C_{B}[0,\infty)$, $M>0$ and $0<\nu\leq1$. The class $\operatorname{Lip}_{M}(\nu) $ is defined as

4.9 $$ \operatorname{Lip}_{M}(\nu)= \bigl\{ f:\bigl\vert f( \zeta_{1})-f(\zeta_{2})\bigr\vert \leq M| \zeta_{1}-\zeta_{2}|^{\nu}\ \bigl( \zeta_{1},\zeta_{2}\in{}[ 0,\infty)\bigr) \bigr\} . $$

### Theorem 4.3

*Let* $D_{n,q}^{\ast}(\cdot ; \cdot)$ *be the operators defined in* (2.2).*Then*, *for each* $f\in \operatorname{Lip}_{M}(\nu)$ ($M>0$, $0<\nu\leq1$) *satisfying* (4.9), *we have*

$$ \bigl\vert D_{n,q}^{\ast}(f;x)-f(x)\bigr\vert \leq M ( \delta_{n,x} ) ^{\frac{\nu}{2}}, $$

*where* $\delta_{n,x}$ *is given in Theorem* 4.1.

### Proof

We prove it by using (4.9) and Hölder’s inequality. We have

$$\begin{aligned} \bigl\vert D_{n,q}^{\ast}(f;x)-f(x)\bigr\vert \leq&\bigl\vert D_{n,q}^{\ast }\bigl(f(t)-f(x);x\bigr)\bigr\vert \\ \leq&D_{n,q}^{\ast} \bigl( \bigl\vert f(t)-f(x)\bigr\vert ;x \bigr) \\ \leq&MD_{n,q}^{\ast} \bigl( \vert t-x\vert ^{\nu};x \bigr) . \end{aligned}$$

Therefore,

$$\begin{aligned}& \bigl\vert D_{n,q}^{\ast}(f;x)-f(x)\bigr\vert \\& \quad \leq M\frac{[n]_{q}}{e_{\mu,q}([n]_{q}r_{[n]_{q}}(x))}\sum_{k=0}^{\infty}\frac{([n]_{q}r_{[n]_{q}}(x))^{k}}{\gamma_{\mu,q}(k)}\biggl\vert \frac{1-q^{2\mu\theta_{k}+k}}{1-q^{n}}-x\biggr\vert ^{\nu} \\& \quad \leq M\frac{[n]_{q}}{e_{\mu,q}([n]_{q}r_{[n]_{q}}(x))}\sum_{k=0}^{\infty } \biggl( \frac{([n]_{q}r_{[n]_{q}}(x))^{k}}{\gamma_{\mu,q}(k)} \biggr) ^{\frac{2-\nu}{2}} \\& \qquad {} \times \biggl( \frac{([n]_{q}r_{[n]_{q}}(x))^{k}}{\gamma_{\mu,q}(k)} \biggr) ^{\frac{\nu}{2}} \biggl\vert \frac{1-q^{2\mu\theta _{k}+k}}{1-q^{n}}-x\biggr\vert ^{\nu} \\& \quad \leq M \Biggl( \frac{n}{e_{\mu,q}([n]_{q}r_{[n]_{q}}(x))}\sum_{k=0}^{\infty } \frac{([n]_{q}r_{[n]_{q}}(x))^{k}}{\gamma_{\mu,q}(k)} \Biggr) ^{\frac{ 2-\nu}{2}} \\& \qquad {} \times \Biggl( \frac{[n]_{q}}{e_{\mu,q}([n]_{q}r_{[n]_{q}}(x))}\sum _{k=0}^{\infty}\frac{([n]_{q}r_{[n]_{q}}(x))^{k}}{\gamma_{\mu ,q}(k)}\biggl\vert \frac{1-q^{2\mu\theta_{k}+k}}{1-q^{n}}-x\biggr\vert ^{2} \Biggr) ^{ \frac{\nu}{2}} \\& \quad = M \bigl( D_{n,q}^{\ast}(t-x)^{2};x \bigr) ^{\frac{\nu}{2}}. \end{aligned}$$

This completes the proof. □

Let

4.10 $$ C_{B}^{2}\bigl(\mathbb{R}^{+}\bigr)=\bigl\{ g\in C_{B}\bigl(\mathbb{R}^{+}\bigr):g^{\prime },g^{\prime \prime} \in C_{B}\bigl(\mathbb{R}^{+}\bigr)\bigr\} , $$

with the norm

4.11 $$ \Vert g\Vert _{C_{B}^{2}(\mathbb{R}^{+})}=\Vert g\Vert _{C_{B}(\mathbb{R}^{+})}+\bigl\Vert g^{\prime}\bigr\Vert _{C_{B}(\mathbb {R}^{+})}+\bigl\Vert g^{\prime\prime}\bigr\Vert _{C_{B}(\mathbb{R}^{+})}, $$

also

4.12 $$ \| g\|_{C_{B}(\mathbb{R}^{+})}=\sup_{x\in\mathbb {R}^{+}}\bigl\vert g(x)\bigr\vert . $$

### Theorem 4.4

*Let* $D_{n,q}^{\ast}(\cdot ; \cdot)$ *be the operators defined in* (2.2). *Then for any* $g\in C_{B}^{2}(\mathbb{R}^{+})$ *we have*

$$ \bigl\vert D_{n,q}^{\ast}(f;x)-f(x)\bigr\vert \leq \biggl\{ \biggl( -\frac {1}{2[n]_{q}} \biggr) +\frac{\delta_{n,x}}{2} \biggr\} \| g \| _{C_{B}^{2}(\mathbb{R}^{+})}, $$

*where* $\delta_{n,x}$ *is given in Theorem* 4.1.

### Proof

Let $g\in C_{B}^{2}(\mathbb{R}^{+})$. Then, by using the generalized mean value theorem in the Taylor series expansion, we have

$$ g(t)=g(x)+g^{\prime}(x) (t-x)+g^{\prime\prime}(\psi)\frac {(t-x)^{2}}{2}, \quad \psi\in(x,t). $$

By applying the linearity property on $D_{n,q}^{\ast}$, we have

$$ D_{n,q}^{\ast}(g,x)-g(x)=g^{\prime}(x)D_{n,q}^{\ast} \bigl( (t-x);x \bigr) +\frac{g^{\prime\prime}(\psi)}{2}D_{n,q}^{\ast} \bigl( (t-x)^{2};x \bigr) , $$

which implies that

$$\begin{aligned}& \bigl\vert D_{n,q}^{\ast}(g;x)-g(x)\bigr\vert \\& \quad \leq \biggl( -\frac {1}{2[n]_{q}} \biggr) \bigl\Vert g^{\prime}\bigr\Vert _{C_{B}(\mathbb{R}^{+})}+ \biggl( [1+2\mu ]_{q}\frac{x}{[n]_{q}}- \frac{1}{4[n]_{q}^{2}} \bigl( 2[1+2\mu]_{q}-1 \bigr) \biggr) \frac{\Vert g^{\prime\prime} \Vert _{C_{B}(\mathbb {R}^{+})}}{2}. \end{aligned}$$

From (4.11) we have $\| g^{\prime}\| _{C_{B}[0,\infty)}\leq\| g\|_{C_{B}^{2}[0,\infty)}$,

$$\begin{aligned}& \bigl\vert D_{n,q}^{\ast}(g;x)-g(x)\bigr\vert \\& \quad \leq \biggl( -\frac {1}{2[n]_{q}} \biggr) \| g\|_{C_{B}^{2}(\mathbb{R}^{+})}+ \biggl( [1+2\mu ]_{q}\frac{x}{[n]_{q}}-\frac{1}{4[n]_{q}^{2}} \bigl( 2[1+2 \mu]_{q}-1 \bigr) \biggr) \frac{\| g\|_{C_{B}^{2}(\mathbb{R}^{+})}}{2}. \end{aligned}$$

The proof follows from (2) of Lemma 2.2. □

The Peetre’s *K*-functional is defined by

4.13 $$ K_{2}(f,\delta)=\inf_{C_{B}^{2}(\mathbb{R}^{+})} \bigl\{ \bigl( \Vert f-g\Vert _{C_{B}(\mathbb{R}^{+})}+\delta\bigl\Vert g^{\prime\prime }\bigr\Vert _{C_{B}^{2}(\mathbb{R}^{+})} \bigr) :g\in\mathcal {W}^{2} \bigr\} , $$

where

4.14 $$ \mathcal{W}^{2}= \bigl\{ g\in C_{B}\bigl( \mathbb{R}^{+}\bigr):g^{\prime},g^{\prime \prime}\in C_{B}\bigl(\mathbb{R}^{+}\bigr) \bigr\} . $$

There exists a positive constant $C>0$ such that $K_{2}(f,\delta)\leq C\omega_{2}(f,\delta^{\frac{1}{2}})$, $\delta>0$, where the second-order modulus of continuity is given by

4.15 $$ \omega_{2}\bigl(f,\delta^{\frac{1}{2}}\bigr)=\sup _{0< h< \delta^{\frac{1}{2}}}\sup_{x\in\mathbb{R}^{+}}\bigl\vert f(x+2h)-2f(x+h)+f(x)\bigr\vert . $$

### Theorem 4.5

*For* $x\geq\frac{1}{2n}$, $n\in\mathbb{N}$ *and* $f\in C_{B}( \mathbb{R}^{+})$, *we have*

$$\begin{aligned}& \bigl\vert D_{n,q}^{\ast}(f;x)-f(x)\bigr\vert \\& \quad \leq2M \biggl\{ \omega_{2} \biggl( f;\sqrt{\frac{ ( -\frac{1}{ [n]_{q}} )+ \delta_{n,x}}{4}} \biggr) +\min \biggl( 1,\frac{ ( -\frac{1}{ [n]_{q}} )+ \delta_{n,x}}{4} \biggr) \| f\| _{C_{B}(\mathbb{R}^{+})} \biggr\} , \end{aligned}$$

*where* *M* *is a positive constant*, $\delta_{n,x}$ *is given in Theorem* 4.3 *and* $\omega_{2}(f;\delta)$ *is the second*-*order modulus of continuity of the function* *f* *defined in* (4.15).

### Proof

We prove this by using Theorem 4.4

$$\begin{aligned} \bigl\vert D_{n,q}^{\ast}(f;x)-f(x)\bigr\vert \leq& \bigl\vert D_{n,q}^{\ast}(f-g;x)\bigr\vert +\bigl\vert D_{n,q}^{\ast}(g;x)-g(x)\bigr\vert +\bigl\vert f(x)-g(x) \bigr\vert \\ \leq& 2 \| f-g \|_{C_{B}(\mathbb{R}^{+})}+ \frac{\delta _{n,x}}{2}\| g \|_{C_{B}^{2}(\mathbb{R}^{+})} + \biggl( -\frac{1}{2 [n]_{q}} \biggr)\| g \|_{C_{B}(\mathbb{R}^{+})}. \end{aligned}$$

From (4.11), clearly, we have $\| g \|_{C_{B}[0,\infty)}\leq\| g \|_{C_{B}^{2}[0,\infty)}$.

Therefore,

$$ \bigl\vert D_{n,q}^{\ast}(f;x)-f(x)\bigr\vert \leq2 \biggl( \| f-g \| _{C_{B}(\mathbb{R}^{+})}+\frac{ ( -\frac{1}{ [n]_{q}} )+ \delta_{n,x}}{4}\| g \|_{C_{B}^{2}(\mathbb{R}^{+})} \biggr), $$

where $\delta_{n,x}$ is given in Theorem 4.1.

By taking infimum over all $g\in C_{B}^{2}(\mathbb{R}^{+})$ and by using (4.13), we get

$$ \bigl\vert D_{n,q}^{\ast}(f;x)-f(x)\bigr\vert \leq2K_{2} \biggl( f;\frac{ ( -\frac{1}{ [n]_{q}} )+ \delta_{n,x}}{4} \biggr). $$

Now, for an absolute constant $Q>0$ in [24], we use the relation

$$ K_{2}(f;\delta)\leq Q\bigl\{ \omega_{2}(f;\sqrt{\delta})+ \min(1,\delta )\| f\|\bigr\} . $$

This completes the proof. □

## Conclusion

The purpose of this paper is to provide a better error estimation of convergence by modification of the *q*-Dunkl analogue of Szász operators. Here we have defined a Dunkl generalization of these modified operators. This type of modification enables better error estimation on the interval $[1/2,\infty)$ if compared to the classical Dunkl-Szász operators [19]. We obtained some approximation results via the well-known Korovkin-type theorem. We have also calculated the rate of convergence of operators by means of modulus of continuity and Lipschitz-type maximal functions.
